# A Randomised Controlled Trial on the Effectiveness and Adherence of Modified Alternate-day Calorie Restriction in Improving Activity of Non-Alcoholic Fatty Liver Disease

**DOI:** 10.1038/s41598-019-47763-8

**Published:** 2019-08-02

**Authors:** Muhammad Izzad Johari, Khairiah Yusoff, Juhara Haron, Chandran Nadarajan, Khairun Nisah Ibrahim, Mung Seong Wong, Muhammad Ilham Abdul Hafidz, Bee Eng Chua, Nurhazwani Hamid, Wan Nor Arifin, Zheng Feei Ma, Yeong Yeh Lee

**Affiliations:** 1 School of Medical Sciences, Universiti Sains Malaysia, Kota Bharu, Malaysia; 2 Hospital Universiti Sains Malaysia, Kota Bharu, Malaysia; 3 Nutrition and Dietetic Unit, Universiti Sains Malaysia, Kota Bharu, Malaysia

**Keywords:** Non-alcoholic fatty liver disease, Nutrition

## Abstract

Currently, there is no effective therapy for non-alcoholic fatty liver disease (NAFLD), although intensive calorie restriction is typically recommended but dietary adherence is an issue. The current study aimed to determine the effectiveness and adherence of eight weeks of modified alternate-day calorie restriction (MACR) in the control of NAFLD activity. This was a randomized controlled trial with MACR as the intervention and normal habitual diet as control. The outcome measures were body mass index (BMI), blood lipids, fasting blood sugar (FBS), liver enzymes (ALT and AST), and ultrasonographic measurements of liver steatosis and shear wave elastography (SWE). Per-protocol (PP) and intention-to-treat (ITT) analysis were performed within and between-groups with P < 0.05 as significant. 43 individuals with NAFLD satisfied study entry criteria, 33 were randomized to MACR and 10 to control group, and, 30 from MACR and 9 from control group completed PP. In between-group analysis of MACR vs. control, BMI were reduced in PP (P = 0.02) and ITT (P = 0.04). Only ALT was reduced in between-group analysis of MACR vs. control, both PP and ITT (P = 0.02 and 0.04 respectively). No reductions in all lipid parameters and FBS were found in between-group analyses (PP and ITT, all P > 0.22). Both liver steatosis grades and fibrosis (SWE) scores were reduced in between-group analyses of MACR vs. controls (PP and ITT, all P < 0.01). Adherence level remained between 75–83% throughout the study. As conclusion, 8 weeks of MACR protocol appears more effective than usual habitual diet in the control of NAFLD activity and with good adherence rate.

## Introduction

Disease activity and progression of non-alcoholic fatty liver disease (NAFLD) to non-alcoholic steatohepatitis (NASH) and cirrhosis can be highly variable, where 3–5% would progress to end-stage liver diseases and mortality in 12.6%^1^. With the rising prevalence of metabolic syndrome and obesity, NAFLD has become the most frequent form of chronic liver disease, not only in the West but also in Asia^2,3^. Currently, there are no approved pharmacological therapies for NAFLD, and many guidelines advocate recommendation with a focus on controlling risk factors and lifestyle changes that include dietary and physical activities^4–6^.

There is good evidence that lifestyle intervention is effective in improving liver histology in NAFLD, for example, Pomrat *et al*. randomised 31 obese patients with NASH into intensive lifestyle changes over 48 weeks versus structured basic education only, and the intensive lifestyle group showed significant improvement in steatosis, necrosis, and inflammation^7^. For dietary intervention, a hypocaloric diet (500–1000 kcal) with 7–10% weight loss target is a recommended strategy in NAFLD^4,5^. Such strategy may be achieved effectively through daily fasting, an extreme form of dietary restriction. Even though such intense restriction has been proven to be effective including prolonging life^8,9^ but some patients find it difficult to adhere and maintain, in addition to potential metabolic adverse effects.

Compared to daily fasting, intermittent fasting (IF) refers to low calorie period lasting less than 24 hours followed by a normal feeding period^8^. IF may achieve more consistent weight loss and improves adherence^10,11^. Modified alternate-day calorie restriction (MACR), the IF strategy employed in our study, restricts 70% of an individual’s daily requirement during restricted day followed by ad-libitum feeding day^12^. There are other different forms of fasting and fasting-mimicking diets which have the potentials to treat NAFLD, although these have not been systematically studied outside of the *in-vitro* environment. For example, periodic fasting (PF) cycles that last 2 or more days separated by a week of normal diet or time-restricted diet which restricts normal calories to only certain hours in a day^13^.

Reported adverse effects associated with IF were minimal such as mild headache or light-headedness and constipation^14^. Other concerns of IF include food craving and compensatory food intake on non-fasting days. However, paradoxically, in the POUNDS LOST trial, food cravings for fats, sweets and starches were reduced with fasting although increased for fruits and vegetables^15^. Likewise, instead of rebound or compensatory eating, IF actually caused a reduction in food intake^16^.

No specific human NAFLD trials have evaluated the effectiveness of MACR in the control of NAFLD activity. Therefore, our study aimed to determine the effectiveness of 8 weeks MACR in improving changes of BMI, biochemical and ultrasound parameters in NAFLD and to evaluate the adherence rate of such dietary strategy.

## Materials and Methods

### Study participants

NAFLD patients who attended the Gastroenterology Clinic at Hospital Universiti Sains Malaysia, a tertiary referral centre for the north-eastern Peninsular Malaysia, were screened from August 2015 till July 2016. To be eligible, participants of either sex were required to have elevated alanine transferase (ALT) and or aspartate transferase (AST) level (ALT > 41 and or AST > 34 IU/L), age that ranged from 18 to 70 years old, BMI between 17.5 and 40 Kg/m^2^ and no evidence of other forms of liver diseases. For those with diabetes mellitus and dyslipidaemia, they must be on a stable therapy for at least 6 months prior to study enrolment. Exclusion criteria included significant alcohol consumption (>1 standard drink per day), pregnancy, and involvement in an active weight loss program or taking weight loss medications, substance abuse and significant psychiatric problems. Withdrawn participants were those unable to tolerate the fasting intervention during the trial and those who dropped-out because of their own choice.

During the first visit, if participants met the selection criteria, informed consent were then taken. Demographic data of participants were recorded. All participants were required to complete a two-week run-in period consisting of maintaining and self-monitoring their usual dietary and daily activities. Participants were given appointment dates within two weeks for anthropometric measurements, ultrasound and bloods for biochemical parameters. Follow-up for participants in the MACR group were at baseline or week 0 (i.e. after completed 2 weeks of run-in), 2-weekly dietitian follow-ups during the 8-week intervention and after completion of intervention at week 8 and for the control group, the visits were at baseline (week 0) and week 8. Anthropometry, ultrasound and bloods were repeated post-intervention for all participants within a week after week 8.

### Study design and sampling

**Figure 1:**
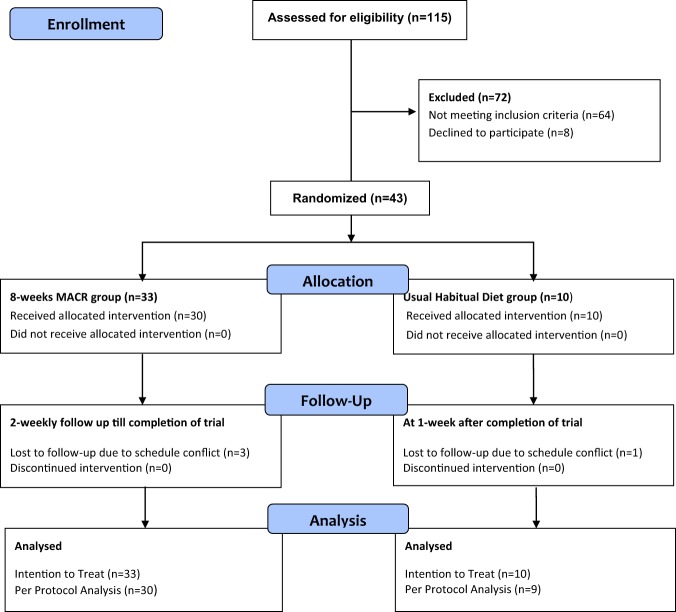
Study flow diagram.

Participants who fulfilled the inclusion and exclusion criteria were randomly assigned to the MACR group or the control group after a 2-week run-in period (Fig. 1).

Random number was generated using the Microsoft Office Excel (Microsoft Corp., Redmond, USA) and randomisation performed with a ratio of three MACR participants to one control participant (3:1). A sample size of 46 participants would achieve a significant difference in studied outcome between intervention and control group using a two-sided test with α = 0.05 and power = 0.8.

### Study interventions

#### Modified alternate-day calorie restriction (MACR) group

**Figure 2:**
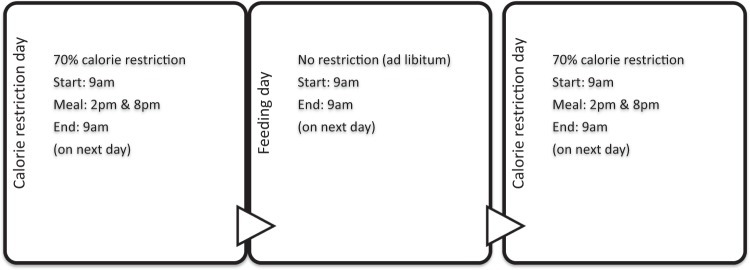
MACR protocol for the 8 weeks duration.

In this group, on fasting day, all participants were instructed to restrict 70% of their calorie requirement per day and on non-fasting day, they ate ad libitum. They were told to eat on the non-fasting day what they normally ate and to the point of satisfaction but not to intentionally overeat. The calorie restriction and feeding days begun at 9am each day, but on fasting day, calorie-deficient meals were only consumed between 2 pm and 8 pm. The MACR protocol is shown in Fig. 2. On fasting day, participants were allowed energy-free beverages, tea, coffee, and sugar-free gum, also encouraged to drink plenty of water. Diet plans were not provided to participants but were self-selected using detailed individualised food portion lists, meal plans, and recipes. To ensure maximal adherence to dietary plan, participants received intermittent phone calls from the investigator (Izzad) and 2-weekly appointments (total four appointments) with a dietitian. Adverse experiences were assessed every 2 weeks until 1 week after the completion of trial.

#### Control group

All participants in the control group would continue their usual habitual diet for 8 weeks. No specific dietary advice was provided throughout the entire trial.

#### Diabetes medications during study period

Previous reports found that insulin sensitizing agents like thiazolidinediones and metformin might induce biochemical and histological effects similar to NAFLD^17^. Therefore, participants, who were already taking thiazolidinedione or metformin, must be on a stable regimen for at least 6 months prior to study enrolment and during the entire study period. Participants were not allowed to start on any of these medications during the study period to avoid any potential confounding effects. Patients who continued to have active liver disease despite on these medications were allowed to participate. If medically necessary, participants were allowed new medications for hyperglycaemia, but the available options only included sulfonylureas, meglitinides and insulin.

### Study procedures and outcomes

#### Body mass index/waist circumference

A standardized calibrated balance (Secca, ZT-120, Hamburg, Germany) was used to measure the weight of participants in light clothing at each study visit. A wall-mounted stadiometer (Secca, ZT-120, Hamburg, Germany) was used to measure height (in m), and together with weight (in Kg), the body mass index (BMI) was calculated. Waist circumference was measured with the participant standing, and with the measuring tape kept horizontal at a level midway between the superior aspect of the iliac crests and the lower lateral margins of the ribs.

#### Blood parameters

Participants were asked to provide 8–10 hours of fasting blood samples at study baseline and at the end of week 8 for biochemical analysis. High-density lipoprotein (HDL), low-density lipoprotein (LDL), triglycerides (TG), total cholesterol, fasting blood sugar and liver enzymes (ALT and AST) were measured.

#### Liver steatosis and fibrosis

**Figure 3:**
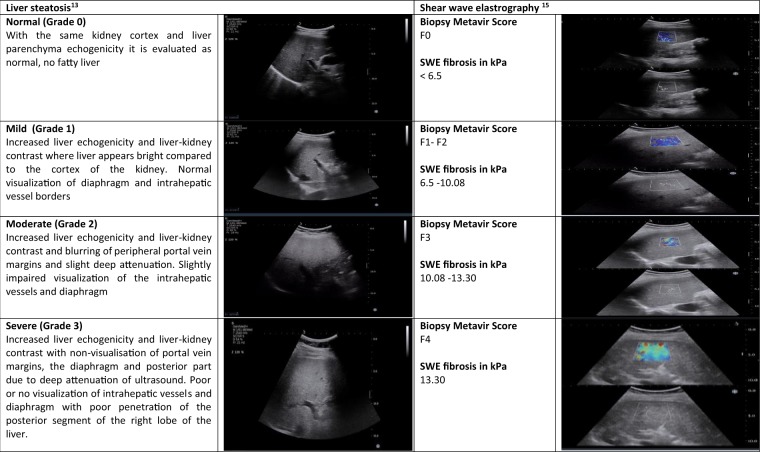
Ultrasonography assessment of steatosis and shear wave elastography of liver.

Ultrasonographic measurements including liver steatosis and 2-dimensional (2D) shear wave elastography (SWE) were performed with the Aixplorer® system (SuperSonic Imagine, Aix-en Provence, France). All measurements of steatosis and 2D SWE were performed by a single sonographer (KY) where the inter-observer agreement with another experienced sonographer (JH) was 85%. Using B-mode, hepatic steatosis was graded as normal (grade 0), mild (grade 1), moderate (grade 2) and severe (grade 3) (Fig. 3)^18^. Based on a recent meta-analysis, ultrasound had a reasonable sensitivity and specificity (84.8% and 93.6% respectively) for steatosis >20%^19^. Using M-mode, 2D SWE was performed according to standards described by the Society of Radiologists in Ultrasound^20^. For each participant, ten consecutive liver stiffness measurements (in kilopascals or kPa) were acquired within the region of interest (ROI) using Q-Box™^21^. Proposed cut-offs of 2D SWE for different METAVIR scores are shown in Fig. 3 ^22^. Recent meta-analysis demonstrated high diagnostic accuracy of liver stiffness measurement with 2D SWE (Area under the receiver operating curve or AUROC of 85.5%)^23^.

#### Assessment of dietary plan adherence

A designated dietitian (KNI) would provide education to all participants regarding calorie restriction and to help monitor their dietary compliance. Adherence to intervention was assessed via a dietary diary and recall. Every two weeks, the dietitian would collect and review the diary. If diary indicated that the participant ate extra calories on a fasting day, that day would be labelled as “not adherent”, but if no extra calories, that day was labelled as “adherent.” Adherence was assessed each week as 1) absolute adherent days and 2) percentage of adherence calculated using the following formula:

$$\frac{{\rm{Number}}\,{\rm{of}}\,{\rm{fast}}\,{\rm{day}}\mbox{'}{\rm{s}}\,{\rm{adherent}}}{{\rm{Number}}\,{\rm{of}}\,{\rm{fast}}\,{\rm{days}}\,{\rm{in}}\,{\rm{week}}}\times 100$$

#### Statistical analyses

Descriptive statistics were computed for all variables; for continuous variable, mean and standard deviation (SD) unless stated otherwise and for categorical variable, frequency and percentage. Distribution was checked using the Kolmogorov-Smirnov analysis and a variable was considered normally distributed if the skewness was within the range of ±2 standard error (SE). Only per-protocol (PP) analysis was performed since the number of participants completing PP and intention-to-treat (ITT) were almost similar. Comparison was made within-group (post- vs. pre-intervention for each randomised group) and between-group (MACR vs. control). Independent t-test and paired t-test were used to compare continuous variables between and within-groups respectively. SPSS version 22 (SPSS Inc., Chicago, United States) was used for all statistical analysis. All *P* values quoted were two-sided with *P* < 0.05 considered as statistically significant.

## Results

### Characteristics of study participants

115 consecutive participants with NAFLD were screened for study entry criteria and 43 participants were found eligible (Fig. 1). Upon randomisation, 33 participants were assigned to the MACR group and 10 participants to the control group. Per-protocol, 30 participants completed MACR and three withdrawn. For the control group, 9 participants completed intervention and one withdrawn due to lost to follow up.

The main characteristics of participants are shown in Table 1. The mean age was 52.60 ± 12.03 and 45.33 ± 10.77 years old for the control group and MACR group, respectively. Male sex was predominant (76.7% or n = 33) and Malays, the major ethnic group (95.3% or n = 41). None of the baseline characteristics showed any significant difference between the two groups (all *P* > 0.05, Table 1). There were 17 participants who were diabetics and 14 of them were on stable metformin but none on thiazolidinedione. Throughout the study, none of the diabetics required dose changes or new medications.

**Table 1:** Baseline characteristics of study participants.

Variables (SD)	Control-group (n = 10) ^a^Mean (SD)	MACR-group (n = 33) ^a^Mean (SD)	*P-value
Age (year)	52.60 (12.03)	45.33 (10.77)	0.59
**Sex**			
Male	9	24	
Female	1	9	0.26
Weight (kg)	78.60	80.78	0.67
BMI (kg/m^2^)	28.21 (3.32)	31.60 (5.19)	0.07
Alanine aminotransferase (U/L)	96.00 (37.39)	83.42 (33.33)	0.37
Aspartate aminotransferase (U/L)	55.60 (18.36)	51.91 (19.47)	0.67
Total cholesterol (mmol/L)	5.09 (1.40)	5.22 (1.24)	0.55
High-density lipoprotein (mmol/L)	3.05 (1.30)	3.40 (0.19)	0.61
Low-density lipoprotein (mmol/l)	1.04 (0.26)	1.15 (0.25)	0.25
Triglycerides (mmol/L)	1.93 (0.94)	1.90 (0.98)	0.89
Fasting blood sugar (mmol/L)	6.89 (2.86)	6.63 (2.09)	0.77
Liver steatosis	2.00 (0.00)	1.91 (0.38)	0.37
SWE (kPa)	6.5 (1.01)	5.9 (1.06)	0.12

Legend; SD, standard deviation; MACR, modified alternate caloric restriction; kg, kilogram; BMI, body mass index; SWE, shear wave elastography; kPa, kilopascal.

^*^P value <0.05 as statistically significant.

^a^Mean at baseline of the trial.

### Anthropometric indexes

**Figure 4:**
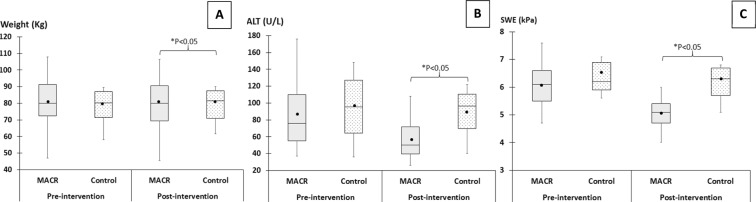
Pre- and post-intervention (MACR vs. control) box plots of (**A**) weight (**B**) ALT level and (**C**) liver fibrosis (shear wave elastography or SWE).

PP results are shown in Table 2. After eight weeks, within the MACR group, there was a significant reduction in mean weight post vs. pre-intervention (78.79 vs. 80.80 Kg, *P* = 0.003) but not within the control group (*P* = 0.86). For between-group analysis, the weight was significantly reduced in MACR vs. control group (mean difference 3.06, 95% CI: 1.14; 4.63, *P* = 0.001) (Fig. 4(A)).

**Table 2:** Changes in physical, biochemical and ultrasonic parameters after 8-weeks of intervention.

Variables	Control Group (Mean)^a^		MACR Group (Mean)^a^		*p-value			Mean difference^c^ (95% CI)
					Within-group^b^		Between-group^c^	
	Pre	Post	Pre	Post	Control	MACR		
Weight (kg)	77.66	78.55	80.80	78.79	0.86	0.003	0.001	3.06 (1.14 to 4.63)
BMI (kg/m^2^)	27.86	28.17	31.73	30.95	0.12	0.003	0.02	1.08 (0.16 to 2.00)
ALT (IU/L)	96.78	90.22	84.33	59.17	0.32	0.001	0.02	18.61(−0.10 to 37.23)
AST (IU/L)	54.22	50.89	51.40	42.77	0.45	0.004	0.34	5.3 (−5.99 to 16.59)
TC (mmol/L)	5.09	5.36	5.32	5.28	0.42	0.78	0.34	0.31 (−0.35 to 0.96)
LDL (mmol/L)	3.19	3.28	3.35	3.36	0.76	0.92	0.75	0.08 (−0.42 to 0.59)
HDL (mmol/L)	1.04	1.11	1.15	1.11	0.31	0.28	1.16	0.12 (−0.05 to 0.29)
TG (mmol/L)	2.02	2.12	1.97	2.09	0.51	0.58	0.97	−0.13 (−0.83 to 0.80)
FBS (mmol/L)	6.85	5.93	6.62	5.87	0.08	0.006	0.74	−0.17 (−1.24 to 0.88)
Liver steatosis	2.00	1.89	1.93	1.43	0.34	0.001	0.01	0.38 (−0.02 to 0.79)
SWE (kPa)	6.57	6.45	5.87	5.01	0.56	0.001	0.01	0.74 (0.19 to 1.29)

Legend; SD, standard deviation; MACR, modified alternate caloric restriction; CI, confidence interval; kg, kilogram; BMI, body mass index; ALT, alanine aminotransferase; AST, aspartate aminotransferase; TC, total cholesterol; LDL, low-density lipoprotein; HDL, high-density lipoprotein; TG, triglycerides; FBS, fasting blood sugar; SWE, shear wave elastography; kPa, kilopascal.

^*^P value  < 0.05 was considered statistically significant ^a^Mean values for pre- and post-intervention for control and MACR group; ^b^Paired *t*-tests; ^c^Independent t-tests.

Likewise, for the MACR group, there was significant reduction in mean BMI post vs. pre-intervention (30.95 vs. 31.73 Kg/m^2^, *P* = 0.003) but not the control group (*P* = 0.12). For between-group analysis, the BMI showed a significant mean reduction in MACR vs. control (mean difference 1.08; 95% CI: 0.16; 2.00, P = 0.02).

### Biochemical indexes

PP results are shown in Table 2. In within-group analysis of the MACR group, there was no reduction post vs. pre-intervention of all lipid parameters (total cholesterol, LDL, HDL and TG; all *P* > 0.28) except for the fasting blood sugar (5.87 vs. 6.62 mmol/L, *P* = 0.006). Likewise, for the control group in within-group analysis, all lipid parameters were not different post vs. pre-intervention (all *P* > 0.31), also for the fasting blood sugar (5.93 vs. 6.85 mmol/L, *P* = 0.08). Similarly, in between-group analysis, the mean difference for all lipid parameters and fasting blood sugar were not statistically significant (all *P* = 0.34) (Table 2).

Within the MACR group, comparing post vs. pre-intervention period, there was a significant reduction of ALT level (59.17 vs. 84.33 IU/L, *P* = 0.001) and likewise, AST level (42.77 vs. 51.40 IU/L, P = 0.004) but not within-group analysis of the control group (*P* > 0.32). In between-group analysis, there was also significant reduction of mean ALT level in MACR vs. control group (mean difference 18.61, 95% CI: −0.10, 37.23, *P* = 0.02) (Fig. 4(B)) however, no difference was reported between the two groups with AST level (*P* = 0.34).

### Liver steatosis and fibrosis (SWE) scores

PP results are shown in Table 2. In within-group analysis of MACR, there was significant reduction post- vs. pre-intervention of liver steatosis grading (1.43 vs. 1.93, *P* = 0.001). Similarly, significant reduction was observed within MACR group of 2D SWE scores (5.01 vs. 5.87, *P* = 0.001). These within-group results were not observed in the control group for steatosis (*P* = 0.34) and SWE scores (*P* = 0.56). In between-group analysis, a statistically significant reduction was observed in the MACR vs. control group, for liver steatosis (mean difference 0.38, 95% CI: −0.02; 0.79, *P* = 0.01) and likewise, for SWE scores (mean difference 0.74, 95% CI: 0.19; 1.29) (Fig. 4(C)).

### Adherence to MACR protocol

**Figure 5:**
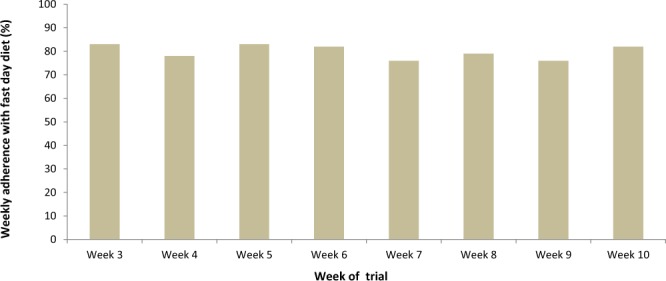
Adherence to MACR protocol.

Figure 5 shows the frequency of participants in the MACR group that were adherent to calorie restriction. No significant drops in adherence levels were observed over the course of 8 weeks with this diet, with levels remained between 75 and 83%. Moreover, no changes were recorded in terms of physical activity habits over the course of the trial; thus, changes in BMI and other disease markers were attributed primarily to the change in diet. No patients receiving intervention discontinued the trial because of difficulty in compliance or adverse events related to MACR.

## Discussion

An age-old practice in many parts of Asia, IF involves periods of voluntary dietary abstinence and it comes in many forms. Ramadan, for example, observed by Muslims worldwide, is a form of IF^24^. In clinical practice guidelines, lifestyle-induced weight loss through dietary restriction and exercises are commonly recommended as initial management of NAFLD^4,5^. However, despite being effective, adherence is a problem^25^. Based on this background, our current trial was aimed to determine the effectiveness and adherence of eight weeks of IF strategy on NAFLD-related markers.

Our employed IF (i.e. MACR) strategy was a form of calorie-restricted regime rather than a strictly time-restricted feeding. By definition, a time-restricted strategy limits calorie intake to a few hours a day but there is no calorie limitation unlike MACR^13^. However, reducing eating hours often results in reduction of daily caloric intake^26^ but not to the extent of 70% that was employed in MACR. Regardless, both strategies can trigger the beneficial fasting physiology, and may be more effective and results in better compliance, when applied together as in MACR.

The study participants were predominantly males, and this was expected where NAFLD are known to be more prevalent among males than females based on published population-based studies^27^. Participants were randomised based on 3:1 ratio to benefit more participants with the fasting intervention. Most patients in the current trial were overweight or obese and this was in concordance with the known observation that NAFLD is prevalent in obese Malaysians especially among the Malay ethnics^28^. There were 17 diabetic participants, but at baseline, both groups had similar proportions of diabetics and similar fasting blood sugar levels. During the entire study, there were no new changes to existing drug regimens of diabetes in both study groups. Despite there being significant changes in within-group analysis of fasting blood sugar in both MACR and control groups, no actual difference was observed in between-group analysis. This indicates that MACR does not compromise the overall control of diabetes and those improvements in biochemical and ultrasonographic parameters observed with MACR are not a result of improvement in diabetes.

This study was able to prove that the IF strategy was effective in improving NAFLD-related biomarkers including weight, BMI and liver transaminases. We observed that weight and BMI decreased with MACR but not in the control group, in both within- and between-group analyses and this occurred despite a stable physical habit. This finding suggests that MACR is effective in controlling weight, albeit to a lesser degree compared to a complete calorie restriction method. There was a mean weight loss of 2.01 kg or 2.5% after MACR. Studies in mice have shown that weight loss lowered visceral fat but without the expense of lean mass loss^29^. A recent systematic review showed that a calorie restrictive diet for 2 weeks to 6 months could achieve a weight loss between 3.0 and 4.9% and led to improvement of liver transaminases in NAFLD^30^.

In our study, we observed a reduction in ALT and AST levels in within-group comparison but only ALT level in between-group analysis. The reduction in liver enzymes might be explained by improvement in visceral fat or steatosis in the liver; an observation substantiated in mice and human^29^. On the other hand, a paradoxical increment of liver enzymes have been observed following fasting however the increment was minimal and reversible upon refeeding^29^. This paradoxical phenomenon may represent a transient autophagy of liver cells from fasting^31,32^ and refeeding allows rapid hepatic regeneration^29^. It is known from literatures that autophagy was impaired in NAFLD^33^ and IF seems to have promoted hepatocyte restorative process in this condition. However, the exact molecular mechanisms that underlie fasting and liver autophagy are unknown but a recent study suggests that PPARα activation from fasting promoted degradation of nuclear receptor co-repressor 1 (NCoR1) and subsequent liver autophagy^34^.

There were no significant changes in all measured lipid parameters (total cholesterol, LDL, HDL and TG) after 8 weeks of MACR protocol. This might be due to our short duration of IF intervention and there was no prescribed exercise regimen. To improve lipids, diet and exercise in combination has been shown to be more effective than diet or exercise alone^35^. On the other hand, a study of 8 weeks IF in asthma has shown a differential changes in lipids with improvement in total cholesterol, TG and HDL but not LDL and glucose^36^. In contrast to the asthma study, our participants had more resistant lipid abnormalities due to obesity and diabetes.

Although liver biopsy is the gold standard, ultrasound is more readily available, cheaper, no or minimal adverse effects and allows measurement of steatosis and fibrosis with reasonable accuracy^19,23^. Steatosis and fibrosis (based on SWE) scores that were used in our study have been previously validated with liver biopsy^18,37^. Furthermore, all ultrasound was performed by a single sonographer, and who had a good agreement level with an expert sonographer. Albeit only 8 weeks and a modified dietary strategy, we have demonstrated a significant improvement of steatosis and fibrosis scores in both within- and between-group analysis after MACR but not in controls. A possible explanation is that majority of study participants had less severe NAFLD with mild to moderate steatosis and fibrosis.

An important limitation of calorie restriction is short and long-term compliance. Our MACR protocol was created to increase short term adherence because the protocol requires energy restriction only every other day^38^. It is also possible to repeat this 8-weekly protocol in cycles rather than continuous over long term. However, such strategy was not tested in our current study. In the present study, we measured the ability of participants to adhere to their fasting day energy goal. Our data showed that adherence to MACR protocol remained between 75–83% throughout the whole eight weeks (Fig. 5). It should be noted that participants also met a designated dietitian every two weeks.

There were other limitations in this trial. Despite being small, the current sample size was adequately powered to achieve study objectives. Many were screened but most did not meet the study criteria and about 10% participants dropped out due to inability to comply with the fasting protocol. Since only few participants were withdrawn during the intervention period, ITT results were not reported. Use of self-reported food diary might lead to under-reporting of calorie intake. Ultrasound has its inherent limitations but nevertheless it was more practical than liver biopsy in actual clinical practice. Other biomarkers including insulin resistance and inflammatory cytokines were not performed in our study, and therefore the exact mechanisms of how such dietary strategy works need further research.

In conclusion, an IF (MACR) strategy may be an effective non-pharmacological strategy to help control activity and progression of mild to moderate NAFLD. It can be considered as an alternative option to intensive daily calorie restriction if adherence is an issue.

### Ethical issues

This study was conducted with the highest respect for participants according to the study protocol, the ethical principles based in the Declaration of Helsinki, and the International Conference and Harmonisation (ICH) - Harmonised Tripartite Guideline for Good Clinical Practice (GCP). All researchers in the study were GCP certified. The current clinical trial was approved by the Human Research and Ethics Committee of Universiti Sains Malaysia (reference: USM/JEPeM/15040117).
