# Author Correction: A Randomised Controlled Trial on the Effectiveness and Adherence of Modified Alternate-day Calorie Restriction in Improving Activity of Non-Alcoholic Fatty Liver Disease

**DOI:** 10.1038/s41598-020-67806-9

**Published:** 2020-06-25

**Authors:** Muhammad Izzad Johari, Khairiah Yusoff, Juhara Haron, Chandran Nadarajan, Khairun Nisah Ibrahim, Mung Seong Wong, Muhammad Ilham Abdul Hafidz, Bee Eng Chua, Nurhazwani Hamid, Wan Nor Arifin, Zheng Feei Ma, Yeong Yeh Lee

**Affiliations:** 1 School of Medical Sciences, Universiti Sains Malaysia, Kota Bharu, Malaysia; 2 Hospital Universiti Sains Malaysia, Kota Bharu, Malaysia; 3 Nutrition and Dietetic Unit, Universiti Sains Malaysia, Kota Bharu, Malaysia

Correction to: *Scientific Reports* 10.1038/s41598-019-47763-8, published online 02 August 2019

The original version of this Article contained errors in the Abstract where,

“Currently, there is no effective therapy for non-alcoholic fatty liver disease (NAFLD), although intensive calorie restriction is typically recommended but dietary adherence is an issue. The current study aimed to determine the effectiveness and adherence of eight weeks of modified alternate-day calorie restriction (MACR) in the control of NAFLD activity. This was a randomized controlled trial with MACR as the intervention and normal habitual diet as control. The outcome measures were body mass index (BMI), blood lipids, fasting blood sugar (FBS), liver enzymes (ALT and AST), and ultrasonographic measurements of liver steatosis and shear wave elastography (SWE). Per-protocol (PP) and intention-to-treat (ITT) analysis were performed within and between-groups with P < 0.05 as significant. 43 individuals with NAFLD satisfied study entry criteria, 33 were randomized to MACR and 10 to control group, and, 30 from MACR and 9 from control group completed PP. In between-group analysis of MACR vs. control, BMI were reduced in PP (P = 0.02) and ITT (P = 0.04). Only ALT was reduced in between-group analysis of MACR vs. control, both PP and ITT (P = 0.02 and 0.04 respectively). No reductions in all lipid parameters and FBS were found in between-group analyses (PP and ITT, all P > 0.22). Both liver steatosis grades and fibrosis (SWE) scores were reduced in between-group analyses of MACR vs. controls (PP and ITT, all P < 0.01). Adherence level remained between 75–83% throughout the study. As conclusion, 8 weeks of MACR protocol appears more effective than usual habitual diet in the control of NAFLD activity and with good adherence rate.”

now reads:

“Currently, there is no effective therapy for non-alcoholic fatty liver disease (NAFLD), and although calorie restriction is recommended in guidelines, but adherence is an issue. The current study aimed to determine the effectiveness of eight weeks intermittent fasting (IF) strategy in the control of NAFLD activity and the adherence rate of such strategy. This was a randomized controlled trial with modified alternate-day calorie restriction (MACR), a form of IF, as the active intervention and usual habitual diet as control. The outcome measures included changes in body mass index (BMI), blood lipids (cholesterol, LDL, HDL and triglyceride), fasting blood sugar (FBS), liver enzymes (ALT and AST), and ultrasound measurements of liver steatosis and 2-dimensional shear wave elastography (SWE). Per-protocol (PP) analysis was performed with comparison within (post vs. pre-intervention) and between (MACR vs. control) groups and P < 0.05 as significant. Of 115 individuals with NAFLD, 43 satisfied the study entry criteria, and 33 were randomised to MACR and 10 to control group, and after 8 weeks, 30 from MACR and 9 from control group completed PP. Significant reduction in weight and BMI (P =0.001 and 0.02 respectively) was observed in MACR vs. control. Likewise, ALT was reduced with MACR but not control (P = 0.02). No reductions in lipid parameters and FBS were found in between-group analyses (all P > 0.22). Both liver steatosis and fibrosis (SWE) scores were significantly reduced in MACR vs. controls (both P < 0.01). Adherence level for MACR remained between 75–83% throughout the study. As a conclusion, eight weeks of IF (MACR) strategy appears more effective than usual habitual diet in the control of NAFLD activity and with good adherence rate.”

These errors have now been corrected in the PDF and HTML versions of the Article.

